# Left Atrial Volume versus Coronary Artery Calcium Score in Patients on Peritoneal Dialysis: An Observational Study

**DOI:** 10.3390/jcm13185539

**Published:** 2024-09-19

**Authors:** Fabrício Moreira Reis, Eduarda Baccarin Ferrari, Nayrana Soares do Carmo Reis, Fabiana Lourenço Costa, Paula Naomi Morimoto, Alejandra Del Carmen Villanueva Maurício, João Carlos Hueb, Rodrigo Bazan, Vanessa Burgugi Banin, Pasqual Barretti, Luis Cuadrado Martin, Silméia Garcia Zanati Bazan

**Affiliations:** 1 Department of Internal Medicine, Botucatu Medical School—UNESP, São Paulo State University, Botucatu 18618-687, Brazil; fabriciomreis@yahoo.com.br (F.M.R.); l.martin@unesp.br (L.C.M.); 2 Department of Neurology, Botucatu Medical School—UNESP, São Paulo State University, Botucatu 18618-687, Brazil

**Keywords:** peritoneal dialysis, coronary artery calcium score, left atrial volume

## Abstract

**Background:** The coronary artery calcium score and left atrial volume have been shown to predict the incidence of acute myocardial infarction and death from cardiovascular disease in patients undergoing peritoneal dialysis. However, the association between these factors has not been well-established. **Methods:** This cross-sectional, prospective, single-center study was conducted on patients undergoing outpatient peritoneal dialysis, who were followed up at a university hospital between March 2018 and August 2019. The coronary artery calcium score was calculated based on cardiovascular computed tomography findings. The score was “positive” when it was ≥100 Agatston and “negative” when it was <100 Agatston. The left atrial volume was obtained using the biplane disc method at the end of the left ventricular systole, and then it was indexed to the body surface. **Results:** Forty-four patients were evaluated. They had an age [mean (range)] of 56 (43–65) years and had been on dialysis therapy for 11.7 (6.8–25.4) months. Univariate analysis revealed a relationship between the coronary artery calcium score and left atrial volume index and the following variables: age, diabetes, overhydration, pulse wave velocity, E/A ratio, and left ventricular mass index. In multivariate logistic regression analysis, only the left atrial volume index was independently associated with a positive coronary artery calcium score. **Conclusions:** The left atrial volume index was associated with a positive coronary artery calcium score in patients on peritoneal dialysis, regardless of other factors. It may be a useful risk marker for coronary artery disease in this population.

## 1. Introduction

Chronic kidney disease (CKD) increases vascular calcification because of the accelerated progression of atherosclerosis and changes in calcium homeostasis [1]. Furthermore, clinical factors such as advanced age, vitamin D therapy, dialysis time, cellular and inflammatory processes, and active osteogenesis in the vascular bed have an important role in this process [2,3,4,5].

Coronary artery disease (CAD) is the major cause of morbidity and mortality in patients with end-stage CKD. These patients are generally asymptomatic until acute myocardial infarction or sudden cardiac death occurs [6]. Coronary calcification, assessed via the coronary artery calcium (CAC) score, which is measured with multidetector computed tomography (CT), is a marker of the atherosclerotic plaque burden, and is useful for predicting the incidence of acute myocardial infarction and death from cardiovascular disease.

In median-aged men without CKD, the diameter of the left atrium has been correlated with the severity of CAD and cardiovascular mortality, with the adjusted risk being 1.2 times for each 5 mm increase in the diameter of the left atrium [7]. Three-vessel CAD is more common than single-vessel CAD in patients with a large left atrium [8]. In patients with dialysis CKD, the left atrial volume (LAV), measured via transthoracic Doppler echocardiography, is independently associated with the presence of silent ischemia [9]. In 2007, researchers in one study [10] evaluated 249 patients on dialysis therapy, of whom 50 patients were on peritoneal dialysis (PD), and demonstrated that a progressive increase in the LAV was a predictor of cardiovascular events in this population.

In the general population, LAV is associated with an elevated CAC score [11], which is a useful marker of advanced CAD [8]. This fact may be mediated by direct ischemia or infarction of the atrium, or by pressure and volume overload secondary to ischemia, and infarction of the left ventricle. Some studies [12,13] have demonstrated that remodeling of the left atrium in patients with stable CAD is predictive of cardiovascular events. In 2017, a study [14] that evaluated patients undergoing hemodialysis revealed an association between the LAV and CAC.

In this context, little evidence exists in the literature regarding the correlation between the LAV and the CAC score in patients with CKD undergoing dialysis, especially the peritoneal modality, and Doppler echocardiography is more accessible than computed tomography in most dialysis services. Thus, the aim of this study was to evaluate this association so that the left atrial volume can emerge as a risk marker for CAD in this population.

## 2. Methods

### 2.1. Study Design

This cross-sectional study included patients with CKD who underwent outpatient PD between March 2018 and August 2019 at the Botucatu Medical School University Hospital, Sao Paulo State University, UNESP; Botucatu, Brazil.

Patients with prevalent PD aged between 18 years and 75 years were included in the study. Individuals who had active or recent infections (<7 days), autoimmune disease, malignant neoplasm, unstable heart disease (e.g., acute coronary syndrome, decompensated heart failure, unstable arrhythmias), previous coronary artery disease, severe left ventricular systolic dysfunction, pericardial effusion, significant mitral or aortic valve disease, primary hyperparathyroidism, calcium supplementation, or who used calcium-based phosphorus binders were not included in this study.

Demographic and clinical data were recorded. The following complementary examinations were conducted with a maximum interval of 2 weeks: biochemical examinations, assessment of nutritional status via anthropometry and bioimpedance bioelectrical impedance analysis (BIA), calculation of residual renal function and dialysis adequacy, Doppler echocardiography, carotid ultrasound, pulse wave velocity (PWV), ankle–brachial index, 24-h ambulatory blood pressure monitoring, and CAC score.

### 2.2. Coronary Artery Calcium Score

CAC scores were calculated using cardiovascular computed tomography (multislice 64-channel Optima scanner; GE Medical Systems, Waukesha, WI, USA). Calcification was defined as a hyperattenuating lesion above a threshold of 130 Hounsfield units (HU) in an area of two or more adjacent pixels observed in the coronary path. The product of the total calcium area and a factor derived from the maximum attenuation (i.e., maximal computed tomographic number) was the calcium score, as described by Agatston et al. [4]; the unit bears his name. The sensitivity and specificity reported in detecting this score are 98.7% and 100%, respectively [15]. The images, including their quality and precision, were analyzed by a single examiner who specialized in cardiovascular tomography and was “blind” to the clinical and laboratory information and echocardiography of the patient. The CAC score was “positive” when it was ≥100 Agatston and “negative” when it was <100 Agatston.

### 2.3. Doppler Echocardiographic Evaluation

The Doppler echocardiogram examinations were conducted by a single expert examiner in the field (who was “blind” to the patient‘s clinical, laboratory, and tomographic information) by using an ultrasound scanner (model HD 15; Philips, Amsterdam, The Netherlands) equipped with a 2.0–5.0 MHz multifrequency ultrasonic transducer and image registration system. Images were obtained and analyzed based on the recommendations of the American Society of Echocardiography [16].

Left ventricular mass (LVM) was calculated using the Devereux [17] formula, and it was indexed to the body surface. The relative thickness of the ventricular wall (2 × posterior wall diastolic thickness/left ventricular [LV] diastolic diameter) was measured to analyze LV geometry based on the description by Ganau et al. [18].

The E and A waves (maximum velocity of rapid and late ventricular filling during atrial contraction, respectively) were obtained via Doppler spectral recording of the transmitral diastolic flow. The measurement of e′ was obtained using spectral tissue Doppler recording of the movement of the middle portion of the mitral annulus. Based on the flow profile and movement of the mitral annulus, the E/A and E/e′ ratios were calculated as markers of LV diastolic function. The LAV was obtained using the biplane disc method, at the end of LV systole. For the analysis, this measurement was indexed to the body surface [19].

### 2.4. Statistical Analysis

A sample size of 47 patients was calculated based on the method described by Baloglu et al. [14], with a correlation coefficient of 0.4, a beta error of 0.2, and an alpha error of 0.05. The included patients were divided into two groups, based on their CAC score, as follows: they were put in the positive group if the score was ≥100 Agatston and the negative group if the score was <100 Agatston. The association between the CAC score and the LAV was evaluated, along with the presence of other CAC markers.

Statistical analyses were conducted using SPSS (version 23.0; SPSS Inc., Chicago, IL, USA). Data were expressed as the frequency, the mean ± standard deviation, or the median (interquartile range), as appropriate. Statistical comparisons between study groups (i.e., between positive and negative calcium scores) were conducted by using the Student’s *t*-test for continuous variables with a normal distribution, the Mann–Whitney test for variables with a non-normal distribution, and the chi-square test for categorical variables. Associations were assessed between the study variables via univariate and multivariate logistic regression models. The positive and negative predictive values, sensitivity, specificity, and accuracy between the LAV and CAC scores were analyzed using the receiver operating characteristic (ROC) curve, with calculation of the area under the ROC curve (AUC). The significance level was set at *p* < 0.05.

### 2.5. Ethical Aspects

This study was approved by the Research Ethics Committee of Botucatu Medical School, São Paulo State University, UNESP (approval no. CAAE 80051517.1.0000.5411). All individuals included in the study signed an informed consent form. All clinical investigation has been conducted according to the principles expressed in the Declaration of Helsinki.

## 3. Results

**Figure 1:**
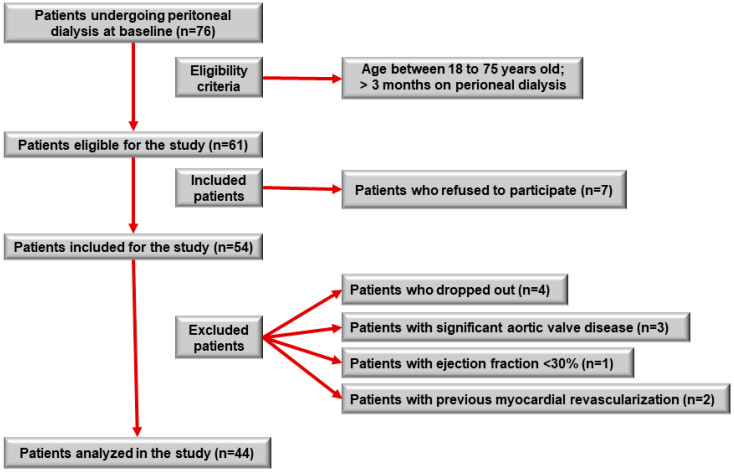
Study participant selection flowchart.

Of the 76 patients who underwent PD during the inclusion period, 61 patients were eligible. Of these, seven patients refused to participate in the study. Fifty-four patients were included; however, four patients withdrew their consent during the study, two patients were excluded because of previous myocardial revascularization, three patients withdrew because of significant aortic valve disease, and one patient withdrew because of an ejection fraction <30% on Doppler echocardiography. Therefore, 44 patients were included in the analysis (Figure 1).

The median age of the patients was 56 years; most patients were male, white, and had low educational levels (i.e., ≤9 years). Most patients were hypertensive and dyslipidemic, with nearly one-half of them having diabetes. Of the patients investigated, 38.6% (17) of patients tested positive for CAC. Compared to patients who tested negative for CAC, patients who tested positive were older (52 years vs. 60 years, *p* = 0.017) and had a higher incidence of diabetes (22.2% vs. 70.6%, *p* = 0.001) (Table 1).

Nearly one-third (31.8%) of all patients had hypertension as the underlying disease of CKD, followed by diabetes (27.3%). The median duration of dialysis therapy was 11.7 months, and the most frequent treatment modality was nocturnal intermittent peritoneal dialysis (NIPD) (54.5%). The groups testing positive and negative for CAC were similar in regard to these variables (Table 2).

In the BIA evaluation, compared to patients who were negative for CAC, patients who were positive for CAC presented with lower mean values for the phase angle (6.5° ± 0.8° vs. 5.5° ± 0.8°, *p* ≤ 0.001), reactance (60.4 ± 10.9Xc vs. 48.6 ± 8.7Xc, *p* ≤ 0.001), and intracellular water (54.5% ± 3.3% vs. 51.1% ± 3.2%, *p* = 0.002); as well as a higher median extracellular water value (45.2% vs. 48.8%, *p* = 0.002); and a higher mean hyperhydration (OH) index value (0.0 ± 1.4l vs. 1.3 ± 1.2l, *p* = 0.002). The other analyzed variables were similar between the groups (Table 3).

Analysis of the laboratory tests revealed that the positive CAC group, compared to the negative CAC group, had a lower HDL value (32.0 mg/dL vs. 39.5 mg/dL, *p* = 0.01), a lower uric acid value (5.6 ± 1.0 mg/dL vs. 6.5 ± 1.5 mg/dL, *p* = 0.038), a higher glycated hemoglobin value (6.2% vs. 5.1%, *p* = 0.010), and a higher alkaline phosphatase value (76.0 U/L vs. 70.5 U/L, *p* = 0.049) (Table 4).

Analysis of the subclinical markers of atherosclerosis revealed that the positive and negative CAC groups were significantly different for femoral PWV (11.4 m/s vs. 8.9 m/s, *p* = 0.025), intima-media thickness of the right carotid artery (0.7 mm vs. 0.6 mm, *p* = 0.037) and left carotid artery (0.7 mm vs. 0.7 mm, *p* = 0.034), and the absence of atherosclerotic plaque in the left carotid artery (46.2% vs. 71.4%, *p* = 0.005) (Table 5).

When comparing the positive CAC and negative CAC groups in relation to ambulatory blood pressure monitoring data, a significant difference was observed only in the nocturnal decrease in systolic pressure (0.4% ± 6.4% vs. 6.0% ± 8.3%, *p* = 0.031) (Table 6).

Analysis of the echocardiographic morphological variables revealed that, compared to the negative CAC group, the positive CAC group had greater values for the thickness of the posterior wall, interventricular septum, and relative wall; as well as the left ventricular mass index (LVMI); the anteroposterior diameter of the left atrium; and the basal diameter of the right ventricle. Among the variables used to evaluate systolic function, only the tricuspid annular plane systolic excursion differed between the groups with mean values, being lower for patients testing positive for CAC than for patients testing negative for CAC (22.6 ± 1.8 mm vs. 24.6 ± 2.0 mm, *p* = 0.002). With regard to diastolic function variables, patients testing positive for CAC, compared to patients testing negative for CAC, had a higher median left atrial volume index (LAVi), based on body surface area (36.1 mL/m^2^ vs. 28.7 mL/m^2^, *p* < 0.001), with lower values for the E wave, A wave, E/A ratio, and e′ septal, e′ lateral, e′ medium, and e′ t waves (Table 7).

Multiple logistic regression was conducted with the predictor variables for positive CAC in the univariate analysis (i.e., age, diabetes, OH index, femoral PWV, LVMI, and E/A ratio), including LAVi. In this evaluation, only the LAVi remained an independent predictor of positive CAC among the patients studied (Table 8).

**Figure 2:**
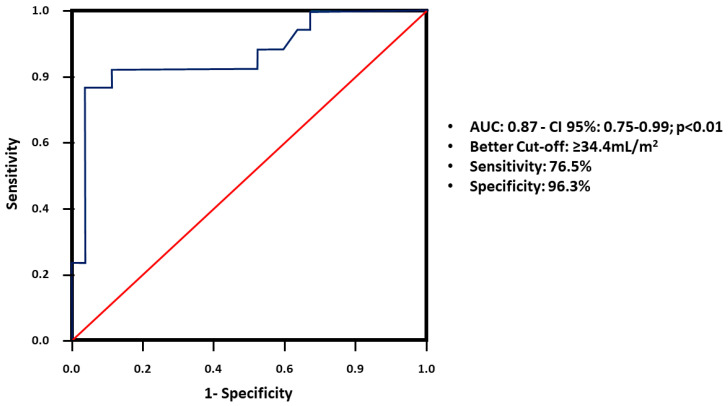
ROC curve for diagnosing positive CAC, based on the LAVi. Abbreviations: ROC, receiver operating characteristic; CAC, coronary artery calcium; LAVi, left atrial volume index; AUC, area under the ROC curve.

In the analysis of the ROC curve for diagnosing positive CAC, based on the LAVi, the best cutoff point was ≥34.4 mL/m^2^. This value had a sensitivity of 76.5% and a specificity of 96.3%, with a positive predictive value of 92.8% and negative predictive value of 86.6% (Figure 2).

## 4. Discussion

The aim of this study was to evaluate this association so that the left atrial volume can emerge as a risk marker for CAD in this population. This study demonstrated an independent association between the LAVi and CAC in patients with PD, even after adjusting for the following variables: age, diabetes, OH index, femoral PWV, LVMI, and E/A ratio.

In the general population, several clinical studies have demonstrated that the size of the left atrium, measured with Doppler echocardiography, is correlated with coronary atherosclerosis [8,14]. In one study, carried out with 100 patients with CAD who underwent coronary cineangiography and Doppler echocardiography, the authors observed that three-vessel coronary disease was much more frequent among individuals who had larger left atria [8]. Laukkanen et al. [7] found a direct and linear relationship between the diameter of the left atrium and the risk of cardiovascular death. They observed that, for each 5 mm increase in diameter of the left atrium, the adjusted risk for cardiovascular death increased by 1.23 times, and that, when the size of the left atrium was greater than 43 mm, patients had a 2.34 times greater risk of cardiovascular death than patients with a normal-sized atrium.

A study by Pan et al. [11], who evaluated 166 patients from the general population who underwent computed tomography of the heart, demonstrated that individuals with higher CAC values (>400 Agatston) had a larger diameter of the left atrium [11]. Baloglu et al. [14], on evaluating 32 patients on hemodialysis, recently found a significant relationship between the size of the left atrium and CAC. The present study demonstrated the same association in patients on PD.

Several pathophysiological links exist between LAV and CAD [20]. First, oxidative stress and endothelial dysfunction [21], which are more pronounced in patients with dialysis kidney disease, are associated with atherosclerosis and can predispose patients to arterial stiffness, thereby causing LV diastolic dysfunction and consequent left atrium (LA) enlargement. Second, the LA is strongly associated with left ventricular hypertrophy [19], a common condition in patients with renal failure, and this fact is associated with abnormal myocardial perfusion [22]. Yameogo et al. [23] found that myocardial ischemia is significantly more frequent in patients with a dilated LA, with increased filling pressure and left ventricular mass. Third, overhydration, which can be measured with the OH index in patients on PD, results in pathological mechanical stimuli in the vascular endothelium and smooth muscle cells, with elevated superoxide production and reduced nitric oxide bioavailability, thereby predisposing patients to atherosclerosis and vascular calcification [10]. Extracellular volume overload also directly contributes to the increase in the LA size.

In this study, when the LAVi was greater than 34.4 mL/m^2^, predicting CAC positivity was possible in 92.8% of cases with a sensitivity of 76.5%, specificity of 96.3%, and accuracy of 88.6%. The LAVi showed a correlation with CAC (>100 Agatston), regardless of diastolic dysfunction, left ventricular mass, arterial stiffness, and fluid overload. This finding indicated that other mechanisms, which could be the subject of future studies, may be involved in this relationship. Therefore, the LAVi can be useful in clinical practice as a marker of CAD risk in patients with PD, regardless of the known traditional risk factors. For example, in patients with few or no traditional risk factors for CAD, but with an enlarged left atrium, we should have more intensive control of comorbidities and greater suspicion regarding the diagnosis of CAD, considering that they have a high risk of significant coronary calcification.

The strengths of this study include the large number of variables analyzed and its prospective design. However, this study had limitations such as the small sample size, although the number of patients was sufficient to identify statistically significant associations. Another limitation is that the study was conducted at a single center; in the future, we intend to subsequently expand our sample size and the external validity of the study in a multicenter study. Furthermore, the cross-sectional observational study design did not allow the establishment of a causal relationship between the LAVi and CAC variables.

These results confirmed the hypothesis that the LAVi, measured with Doppler echocardiography, was associated with a positive CAC score, measured via computed tomography among patients with chronic kidney disease undergoing PD. Therefore, considering that Doppler echocardiography is a more accessible and noninvasive test performed routinely for patients on PD in many services, using the LAVi as a marker of risk for coronary artery disease is feasible for this population.

## Figures and Tables

**Table 1:** Demographic and clinical characteristics of the study participants.

	Total (*n* = 44)	Patients with a Positive Calcium Score (*n* = 17)	Patients with a Negative Calcium Score (*n* = 27)	*p*
Age (y)	56.0 (43.0–65.0)	60.0 (56.0–67.0)	52.0 (38.0–62.0)	0.017
Sex, male (%)	54.5	74.7	48.1	0.283
Skin color, white (%)	65.9	76.5	59.3	0.260
Education, ≤9 years of study (%)	63.7	58.8	66.6	0.721
Income, 2–4 MW (%)	56.8	58.8	55.6	0.632
DM (%)	40.9	70.6	22.2	0.001
SAH (%)	81.8	88.2	77.8	0.381
Dyslipidemia (%)	79.5	82.4	77.8	0.714
BMI (kg/m^2^)	27.5 ± 4.8	27.8 ± 4.6	27.4 ± 5.0	0.766
Family history of CAD (%)	22.9	36.4	16.7	0.198
Smoking (%)	9.1	17.6	3.7	0.117
Alcohol intake (%)	9.1	5.9	11.1	0.557
Physical activity (% sedentary)	70.5	70.6	70.4	0.231

Data are expressed as the mean ± the standard deviation, the median (interquartile range), or the frequency. Student‘s *t*-test was used for comparisons of continuous variables; the chi-square test was used to compare categorical variables. Abbreviations: MW, minimum wage; DM, diabetes mellitus; SAH, systemic arterial hypertension; BMI, body mass index; CAD, coronary artery disease.

**Table 2:** Dialysis data of the study participants.

	Total (*n* = 44)	Patients with a Positive Calcium Score (*n* = 17)	Patients with a Negative Calcium Score (*n* = 27)	*p*
Time in PD (mo.)	11.7 (6.8–25.4)	10.2 (6.7–19.2	12.9 (6.6–26.7)	0.372
Basic disease				
DM (%)	27.3	35.3	22.2	0.224
SAH (%)	31.8	23.5	37.0	
Glomerulopathies (%)	20.5	11.8	25.9	
Other (%)	20.4	29.4	14.9	
Previous HD (%)	13.6	17.6	11.1	0.538
PET glucose classification (% HM)	50.0	58.8	44.4	0.641
PET creatinine classification (% LM)	54.5	58.8	51.9	0.651
Total weekly Kt/V	2.2 (1.7–2.4)	2.2 (1.7–2.3)	2.2 (1.7–2.7)	0.484
Urine output (mL)	1000.0 (441.2–1500.0)	900.0 (355.0–1500.0)	1000.0 (382.5–1500.0)	0.857
Dialysis modality (% NIPD)	54.5	52.9	55.6	0.861

Data are expressed as the median (interquartile range) or as the frequency. Student’s *t*-test, Mann–Whitney *U*-test, or chi-square test. Abbreviations: PD, peritoneal dialysis; DM, diabetes mellitus; SAH, systemic arterial hypertension; HD, hemodialysis; PET, peritoneal equilibrium test; HM, high medium; LM, low medium; Kt/V, dialysis adequacy; NIPD, nocturnal intermittent peritoneal dialysis.

**Table 3:** Bioimpedance variables among the study participants.

	Total (*n* = 44)	Patients with a Positive Calcium Score (*n* = 17)	Patients with a Negative Calcium Score (*n* = 27)	*p*
Phase angle (°)	6.1 ± 0.9	5.5 ± 0.8	6.5 ± 0.8	<0.001
Resistance (Ohm)	521.3 ± 93.8	500.7 ± 89.3	534.2 ± 95.9	0.253
Reactance (Xc)	55.8 ± 11.6	48.6 ± 8.7	60.4 ± 10.9	<0.001
Body cell mass (kg)	23.3 (18.4–27.1)	23.2 (18.0–27.8)	23.6 (18.8–27.2)	0.718
Lean mass (%)	70.0 ± 7.2	69.7 ± 5.8	70.2 ± 8.0	0.842
Fat mass (%)	30.0 ± 7.2	30.3 ± 5.8	29.8 ± 8.0	0.841
Intracellular water (%)	53.2 ± 3.6	51.1 ± 3.2	54.5 ± 3.3	0.002
Extracellular water (%)	47.1 (44.1–49.1)	48.8 (46.7–50.7)	45.2 (43.0–48.1)	0.002
Total body water (L)	36.0 (30.4–42.9)	38.5 (27.7–48.8)	35.6 (30.7–42.8)	0.539
OH index (L)	0.5 ± 1.5	1.3 ± 1.2	0.0 ± 1.4	0.002

Data are expressed as the mean ± the standard deviation or as the median (interquartile range). Student‘s *t*-test was used for comparisons of continuous variables; the chi-square test was used to compare categorical variables. Abbreviation: OH, hyperhydration.

**Table 4:** Laboratory test results of the study participants.

	Total (*n* = 44)	Patients with a Positive Calcium Score (*n* = 17)	Patients with a Negative Calcium Score (*n* = 27)	*p*
Hemoglobin (g/dL)	11.6 ± 1.1	11.7 ± 1.0	11.2 ± 1.2	0.549
Urea (mg/dL)	103.7 ± 22.9	110.3 ± 23.8	99.5 ± 21.8	0.131
Creatinine (mg/dL)	9.0 ± 3.0	8.2 ± 2.6	9.4 ± 3.2	0.207
Corrected calcium (mg/dL)	9.2 ± 0.8	9.0 ± 0.7	9.3 ± 0.8	0.280
Magnesium (mmol/L)	1.9 (1.8–2.3)	1.9 (1.8–2.2)	1.8 (1.6–2.2)	0.528
Phosphorus (mg/dL)	5.2 ± 4.9	5.5 ± 1.1	5.1 ± 1.6	0.327
HDL (mg/dL)	37.5 (31.0–47.2)	32.0 (30.0–34.0)	39.5 (35.0–54.5)	0.015
LDL (mg/dL)	78.0 (59.2–99.4)	60.0 (32.0–82.6)	74.6 (53.0–88.2)	0.737
Triglycerides (mg/dL)	153.5 (117.2–207.0)	230.0 (154.0–287.0)	133.0 (101.0–192.5)	0.065
Total cholesterol (mg/dL)	151.5 (123.5–183.2)	136.0 (113.0–161.0)	153.0 (108.2–181.7)	0.745
Glycated hemoglobin (%)	5.5 (5.1–6.4)	6.2 (5.5–8.8)	5.1 (5.0–5.3)	0.010
Albumin (g/dL)	3.7 ± 0.4	3.7 ± 0.4	3.6 ± 0.4	0.645
Uric acid (mg/dL)	6.1 ± 1.4	5.6 ± 1.0	6.5 ± 1.5	0.038
Alkaline phosphatase (U/L)	84.5 (68.2–135.0)	76.0 (67.0–155.0)	70.5 (60.5–79.5)	0.049
Ultrasensitive CRP (mg/dL)	1.2 (0.7–7.5)	7.5 (0.9–9.1)	0.9 (0.0–2.1)	0.232
PTH (mg/dL)	245.0 (171.7–363.5)	339.0 (145.0–377.0)	184.0 (161.2–580.7)	0.151
Vitamin D (ng/mL)	25.7 (19.9–30.8)	25.4 (19.9–27.2)	24.6 (18.1–28.4)	0.587

Data are expressed as the mean ± the standard deviation or as the median (interquartile range). Student‘s *t*-test or Mann–Whitney *U*-test. Abbreviations: HDL, high-density lipoprotein; LDL, low-density lipoprotein; CRP, C-reactive protein; PTH, parathyroid hormone.

**Table 5:** Subclinical markers of atherosclerosis among the study participants.

	Total	Patients with a Positive Calcium Score	Patients with a Negative Calcium Score	*p*
Radial PWV (m/s) ^1^	7.9 (7.1–8.7)	8.6 (7.4–9.2)	7.7 (6.4–8.5)	0.219
Femoral PWV (m/s) ^1^	9.0 (7.2–12.4)	11.4 (8.3–13.2)	8.9 (6.8–11.2)	0.025
Central mean SBP (mm/Hg) ^1^	117.6 ± 16.6	113.2 ± 16.1	121.6 ± 16.3	0.130
Central SBP (mm/Hg) ^1^	130.1 ± 16.8	128.4 ± 20.4	131.6 ± 13.2	0.581
Central DBP (mm/Hg) ^1^	95.7 ± 13.2	92.6 ± 12.9	98.5 ± 13.2	0.188
ABI ^2^	1.1 (1.0–1.1)	1.0 (0.8–1.1)	1.1 (1.0–1.1)	0.708
CIMT right (mm) ^3^	0.6 (0.6–0.7)	0.7 (0.6–0.9)	0.6 (0.5–0.7)	0.037
CMIT left (mm) ^3^	0.7 (0.6–0.8)	0.7 (0.6–1.0)	0.7 (0.6–0.7)	0.034
Atheroma plaques on the right ^3^				
Absent (%)	52.9	38.5	61.9	0.325
Type 1 (%)	0.0	0.0	0.0	
Type 2 (%)	2.9	0.0	4.8	
Type 3 (%)	14.9	15.4	14.3	
Type 4 (%)	29.4	46.2	19.0	
Atheroma plaques on the left ^3^				
Absent (%)	61.8	46.2	71.4	0.005
Type 1 (%)	0.0	0.0	0.0	
Type 2 (%)	8.8	7.7	9.5	
Type 3 (%)	11.8	0.0	19.0	
Type 4 (%)	17.6	46.2	0.0	

Data are expressed as the mean ± the standard deviation, median (interquartile range), or frequency. Student‘s *t*-test was used for comparisons of continuous variables; the chi-square test was used to compare categorical variables. ^1^ The total number of patients is 35: for the CAC^+^ group, *n* = 17, and CAC^−^ group, *n* = 18. ^2^ The total number of patients is 44: for the CAC^+^ group, *n* = 17, and CAC^−^ group, *n* = 27. ^3^ The total number of patients is 36: for the CAC^+^ group, *n* = 13, and CAC^−^ group, *n* = 23. Abbreviations: CIMT, carotid intima-media thickness; ABI, ankle–brachial index; DBP, diastolic blood pressure; SBP, systolic blood pressure; PWV, pulse wave velocity.

**Table 6:** Ambulatory blood pressure monitoring data of the study participants.

	Total (*n* = 41)	Patients with a Positive Calcium Score (*n* = 15)	Patients with a Negative Calcium Score (*n* = 26)	*p*
24 h SBP (mm/Hg)	130.2 ± 15.6	133.1 ± 12.6	128.6 ± 17.1	0.386
24 h DBP (mm/Hg)	79.2 ± 11.2	79.7 ± 8.0	78.8 ± 12.8	0.810
24 h MAP (mm/Hg)	92.0 (86.5–105.0)	92.0 (90.0–105.0)	92.5 (81.7–107.2)	0.445
Waking SBP (mm/Hg)	131.3 ± 15.7	133.0 ± 12.6	130.3 ± 17.4	0.604
Waking DBP (mm/Hg)	80.2 ± 11.6	79.9 ± 8.1	80.4 ± 13.3	0.902
Waking MAP (mm/Hg)	96.4 ± 12.4	97.0 ± 8.8	96.1 ± 14.2	0.824
Sleep SBP (mm/Hg)	126.0 ± 17.4	132.4 ± 14.1	122.3 ± 12.3	0.073
Sleep DBP (mm/Hg)	74.5 ± 11.4	77.3 ± 8.8	72.9 ± 12.6	0.238
Sleep MAP (mm/Hg)	90.0 ± 12.3	93.4 ± 9.9	88.0 ± 13.3	0.179
Night SBP descent (%)	3.9 ± 8.0	0.4 ± 6.4	6.0 ± 8.3	0.031
Night DBP descent (%)	6.8 ± 9.8	2.9 ± 9.9	9.0 ± 9.2	0.055
Pulse pressure (mm/Hg)	48.9 (42.4–55.0)	53.0 (45.4–55.4)	48.4 (43.1–53.6)	0.455

Data are expressed as the mean ± the standard deviation or as the median (interquartile range). Student‘s *t*-test was used for comparisons of continuous variables; the chi-square test was used to compare categorical variables. Abbreviations: SBP, systolic blood pressure; DBP, diastolic blood pressure; MAP, mean arterial pressure.

**Table 7:** Doppler echocardiographic variables of the study participants.

	Total (*n* = 44)	Patients with a Positive Calcium Score (*n* = 17)	Patients with a Negative Calcium Score (*n* = 27)	*p*
Morphological variable				
LVDD (mm)	48.7 ± 3.8	49.5 ± 3.6	48.3 ± 3.9	0.328
LVSD (mm)	30.0 (27.0–31.0)	30.0 (28.5–32.0)	29.0 (26.0–31.0)	0.321
PW (mm)	11.0 (10.0–12.0)	12.0 (11.5–13.0)	11.0 (9.0–12.0)	0.001
IVS (mm)	12.0 (11.0–13.0)	13 (12.0–13.5)	11.0 (9.0–12.0)	<0.001
RWT	0.5 ± 0.1	0.5 ± 0.1	0.4 ± 0.1	0.005
LVM (g)	213.2 ± 53.9	249.4 ± 48.7	190.3 ± 44.2	<0.001
LVMI (g/m^2^)	117.5 ± 25.8	133.5 ± 20.8	107.4 ± 23.7	0.001
LA (mm)	41.1 ± 4.8	44.4 ± 4.1	40 ± 3.9	<0.001
RV (mm)	33.8 ± 2.9	34.9 ± 2.5	33.1 ± 2.9	0.047
Systolic function variable				
%deltaD	0.4 (0.4–0.4)	0.4 (0.4–0.4)	0.4 (0.4–0.4)	0.423
EF (%)	66.9 (64.9–70.9)	68.0 (65.1–71.5)	66.0 (64.9–69.4)	0.252
Mean S (cm/s)	8.6 ± 1.0	8.5 ± 0.8	8.7 ± 1.1	0.639
S t (cm/s)	12.5 ± 1.6	12.0 ± 1.5	12.9 ± 1.5	0.060
TAPSE (mm)	23.8 ± 2.1	22.6 ± 1.8	24.6 ± 2.0	0.002
CO (L/min)	5.5 ± 0.7	5.4 ± 0.8	5.5 ± 0.6	0.556
Diastolic function variable				
LAVi (mL/m^2^)	29.8 (27.6–35.6)	36.1 (33.6–38.6)	28.7 (23.7–30.1)	<0.001
E (cm/s)	80.1 ± 18.9	71.9 ± 15.7	85.2 ± 19.2	0.022
A (cm/s)	92.5 (76.0–107.5)	102.0 (86.5–109.5)	87.0 (67.0–106.0)	0.029
E/A	0.8 (0.7–1.4)	0.7 (0.6–0.8)	0.8 (0.7–1.5)	0.003
e′ septal (cm/s)	6.0 (5.4–8.2)	5.6 (5.1–6.1)	7.1 (5.8–10.2)	0.01
e′ lateral (cm/s)	7.6 (6.3–10.5)	7.0 (5.9–7.8)	8.6 (7.2–12.8)	0.011
e′ medium (cm/s)	6.8 (5.7–9.1)	6.3 (5.5–6.8)	8.2 (6.5–11.2)	0.003
E/e′ medium	10.8 ± 2.5	11.6 ± 2.5	10.3 ± 2.5	0.102
e′ t (cm/s)	8.7 (7.8–10.3)	7.9 (6.8–8.9)	9.4 (8.5–10.4)	0.002
a′ t (cm/s)	12.1 ± 2.4	11.7 ± 1.8	12.4 ± 2.7	0.357

Data are expressed as the mean ± the standard deviation or as the median (interquartile range). Student′s *t*-test or Mann–Whitney *U*-test were used. Abbreviations: LVDD, left ventricular diastolic diameter; LVSD, left ventricular systolic diameter; PW, posterior wall diastolic thickness; IVS, interventricular septal diastolic thickness; RWT, relative wall thickness; LVM, left ventricular mass; LVMI, left ventricular mass index; LA, left atrial diameter; RV, right ventricular; %deltaD, left ventricular shortening fraction; EF, left ventricular ejection fraction by the Simpson method; Mean S, mitral annulus systolic excursion velocity on tissue Doppler (average of medial and lateral portions); St, tricuspid annulus systolic excursion velocity on tissue Doppler; TAPSE, tricuspid annular plane systolic excursion; CO, cardiac output; LAVi, left atrial volume index; E, transmitral flow peak velocity in the rapid ventricular filling phase; A, transmitral flow peak velocity during the atrial contraction phase; e′, diastolic excursion velocity of the mitral annulus on tissue Doppler; e′ t, diastolic excursion velocity of the tricuspid annulus on tissue Doppler; a′ t, diastolic excursion velocity of the tricuspid annulus on tissue Doppler.

**Table 8:** Multivariate logistic regression with variables predictive of CAC positivity among the study patients.

Variable	B	OR	*p*	Confidence Interval	
				Inferior	Superior
Age	0.04	1.04	0.55	0.92	1.17
DM	0.07	0.93	0.96	0.04	20.95
OH	−0.04	0.96	0.93	0.36	2.56
Femoral PWV	0.07	1.07	0.71	0.74	1.56
LVMI	0.01	1.01	0.66	0.95	1.08
E/A	1.48	4.1	0.57	0.26	737.64
LAVi	0.53	1.71	0.03 ^a^	1.06	2.75

Multivariate logistic regression analysis model: age, DM, OH, femoral PWV, LVMI, E/A, and LAVi. ^a^ *p* < 0.05. Abbreviations: CAC, coronary artery calcification; OR, odds ratio; DM, diabetes mellitus; OH, hyperhydration index; PWV, pulse wave velocity. LVMI, left ventricular mass index; E, transmitral flow peak velocity in the rapid ventricular filling phase; A, transmitral flow peak velocity during the atrial contraction phase; LAVi, left atrial volume index.

## Data Availability

The datasets presented in this study can be found in online repositories. The names of the repository/repositories and accession number(s) can be found below: http://hdl.handle.net/11449/215612 (accessed on 24 August 2024).
